# Nanoparticles Induced Oxidative Damage in Reproductive System and Role of Antioxidants on the Induced Toxicity

**DOI:** 10.3390/life13030767

**Published:** 2023-03-13

**Authors:** Antony V. Samrot, Lawrence Xavier Noel Richard Prakash

**Affiliations:** 1 School of Bioscience, Faculty of Medicine, Bioscience and Nursing, MAHSA University, Jalan SP2, Bandar Saujana Putra, Jenjarom 42610, Malaysia; 2 Department of Biotechnology, School of Bio and Chemical Engineering Sathyabama Institute of Science and Technology, Chennai 600119, Tamil Nadu, India; noelrichardp@gmail.com

**Keywords:** antioxidant, reproductive system, nanotoxicity

## Abstract

Nanotechnology is used in a variety of scientific, medical, and research domains. It is significant to mention that there are negative and severe repercussions of nanotechnology on both individuals and the environment. The toxic effect of nanoparticles exerted on living beings is termed as nanotoxicity. Nanoparticles are synthesized by various methods such as chemical, biological, physical, etc. These nanoparticles’ nanotoxicity has been observed to vary depending on the synthesis process, precursors, size of the particles, etc. Nanoparticles can enter the cell in different ways and can cause cytotoxic effects. In this review, the toxicity caused in the reproductive system and the role of the antioxidants against the nanotoxicity are briefly explained.

## 1. Introduction

Nanotechnology is an emerging field that focuses on the design, characterization, manufacturing of devices, and systems by manipulating shape and size at the nanoscale [1]. Recent developments in nanotechnology and nanoscience find its application in various fields such as food technology, medicine, transportation, drug delivery, cosmetics, etc. [1,2,3,4,5,6]. Nanomaterials can interact with cells and the milieu in a way that their chemically identical, larger biological counterparts cannot, and this is because of their exceedingly small size [7]. When nanoparticles are discharged into the environment, they may readily enter the cells by receptor-mediated endocytosis or by passive diffusion, where they interact with the cellular proteins, lipids, and genomic DNA [8,9]. This results in oxidative stress caused by Reactive Oxidative Species which is considered to be the most significant contributor to nanotoxicity [10,11]. Despite the antioxidant property of a few nanoparticles like gold, silver, copper, iron nanoparticles, etc., most of them are involved in the formation of intracellular ROS depending on the cellular absorption of nanoparticles, intracellular response, and intracellular metal ion release [12,13,14] and are produced during several cellular signalling processes as well as they are a part of the immune system’s defense mechanism. ROS damages biological macromolecules such as proteins, lipids, and DNA, causing negative effects on cells and causing mitochondrial dysfunction [15]. A high level of ROS can also produce a range of physiopathological effects, such as apoptosis, necrosis, hypertrophy, genotoxicity, inflammation, fibrosis, and even cancer. It also increases the production of pro-inflammatory cytokines and activates inflammatory cells like macrophages, which further increases the production of ROS [16]. These nanoparticles could also affect the reproductive system by breaching the guard tissues of the reproductive system like the epithelial, blood-testis, placenta and placental barriers and reproductive cells like germ cells, Leydig cells, and Sertoli cells [17]. Particularly, in the male reproductive system, nanoparticles could cause toxic effects by creating ROS which acts as molecular mediators in signal transduction pathways of spermatogenesis, steroidogenesis, and hypothalamic-pituitary-gonadal axis regulation affecting sperm maturation, such as DNA compaction and flagellar modification; whereas, in female reproduction, nanoparticles affect the physiological processes, including maturation of the oocyte, fertilization, development of the embryo, and pregnancy [18,19]. The prevalence of interspecies extrapolation factors between humans and test species helps in computing the toxicology in the reproductive system after exposure to drugs or chemicals [18]. Thus, this review briefs readers on the impacts of nanoparticles on the reproductive system, its mechanism, quantitative test, and potential nanotoxicity, using animal models.

## 2. Nanoparticles and Reactive Oxygen Species (ROS)

**Figure 1:**
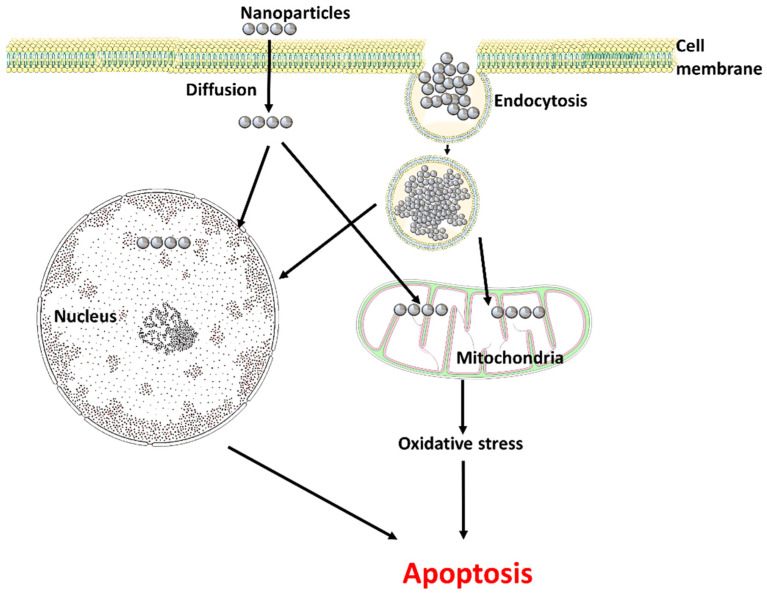
Entry of nanoparticles into the cell (Figure was drawn using the images and illustrations available on Smart Servier Medical Art).

The unique characteristics of nanoparticles make them suitable for a variety of applications, such as biomedical applications, chemical and biological sensors, drug delivery, and various other fields [20]. The physical features of nanoparticles have a significant impact on cellular interactions. Nanoparticles can enter cells via endocytosis, diffusion, or via interactions with phospholipids. They can disrupt cell membranes by physicochemical interactions with the surface of the membrane, thereby impairing transport processes. Additionally, they can also trigger oxidative stress by releasing ions. Also, the function of cell organelles, particularly the mitochondria and peroxisomes, can be impacted by nanoparticles, which can disrupt intracellular transport and cause oxidative stress [21] (Figure 1).

**Figure 2:**
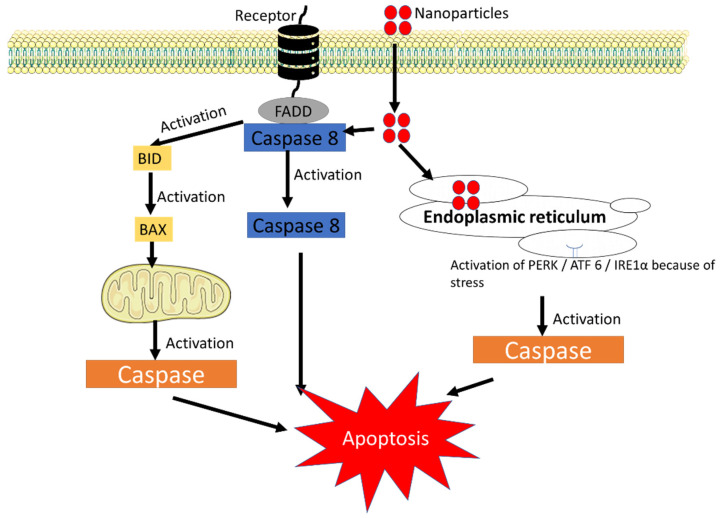
Nanoparticles interfering with the cell signalling pathway to induce apoptosis. ATF6, activating transcription factor 6; PERK, protein kinase R-like ER kinase IRE1α, inositol-requiring enzyme 1α (Figure was drawn using the images and illustrations available on Smart Servier Medical Art).

Silver, platinum, cerium, and zinc nanoparticles are some of the metal-based nanoparticles that can cause membrane lipid peroxidation and oxidative stress leading to mitochondrial dysfunction, DNA damage, and a number of other adverse effects [22]. Hydrogen peroxide, hydroxyl radicals, singlet oxygen, and superoxide anion radicals are some examples of biologically relevant ROS produced by normal cellular metabolism or by any antigenic/mitogenic responses or as a by-product in mitochondrial respiration [23]. Also, ROS are generated when immune cells like neutrophils and macrophages react against environmental toxins/microorganisms/any other antigens/any internal stimuli [24]. Oxidative stress occurs when ROS are abundant in the body and cause potentially harmful biological reactions. This is because ROS produces an imbalance between the amount produced and the biological system’s ability to quickly detoxify reactive intermediates or to repair the damage by activating antioxidant enzymes and non-enzymatic antioxidants [24,25]. When the ROS level exceeds the level because of nanoparticle entry, immune cell activation and activated cell signalling pathways lead to additional pathological effects such as inflammation, genotoxicity, and fibrosis [24]. Most nanoparticles could interfere with the cell signalling pathway through three primary routes resulting in the apoptotic process (Figure 2). The direct nanoparticle occupancy of the FADD receptor is the first mechanism. The death receptor including TNFR1 (TNF receptor superfamily member 1A), FAS/CD95 (FAS cell surface death receptor) etc. could initiate death signalling, where the ligands bind to these receptors and activate FADD (FAS associated via death domain) and then activate proCASP8 to CASP8 and end with activating apoptosis [26]. The second pathway involves the location of nanoparticles pacing in the endoplasmic reticulum; while the endoplasmic reticulum is facing high stress, there is the activation of Protein kinase R-like ER kinase (PERK), inositol-requiring enzyme 1α (IRE1α), and activating transcription factor 6 (ATF6), which are important transmembrane proteins that cascade various cellular signalling pathways, ending with activation of caspase and leading to apoptosis [27,28,29]. The third pathway involves modulating the mitochondrial function in the presence of nanoparticles. All these mechanisms eventually lead to caspase activation, which causes the mitochondria to produce more ROS, to make more BH3 Interacting Domain Death Agonist (BID protein), and to activate Bax or Bak1 proteins, all of which can cause cell death, DNA cleavage, and organelle damage [30]. The mechanism by which each nanoparticle produces ROS is unique; to date, there is no exact evidence that shows how the ROS are produced. Through Fenton-type reactions, metal-containing nanoparticles cause toxicity by producing free radicals, whereas carbon nanotube-mediated ROS production is significantly influenced by mitochondrial injury [31]. Also, nanoparticles alter electron transfer, elevating the ratio of NADP^+^/NADPH, and interfering with mitochondrial function, resulting in intracellular ROS formation. Additionally, nanoparticles are known to affect genes associated with oxidative stress, such as *oxyR*, *ahpC*, *soxR*, and *soxS* antioxidant genes such as *gpx*, *sod1*, and NADPH synthesis genes such as met9. Nanoparticles are associated with an increase in intracellular ROS because of their instability in oxidative and antioxidant defence genes [16]. Based on the mechanism, nanotoxicity is classified into two types: (i) primary oxidative stress, and (ii) secondary oxidative stress. Primary oxidative stress is defined as the photocatalytic activity of metal nanoparticles, for example titanium oxide could directly induce oxidative stress by ROS production, whereas secondary oxidative stress causes mitochondrial malfunction by the exposure to nanoparticles that results in the formation of ROS [12].

## 3. Entry of Nanoparticles into the Reproductive System

Since the 20th century, experiments in consumer items, industry, and medicine have significantly increased, which also increases the number of nanosized particles in the air. This increase in the air holds the chance of nanoparticle-induced toxicity in humans [32,33,34]. Generally, the entry of nanoparticles into the human body is comprised of four types: (i) ingestion; (ii) inhalation; (iii) dermal penetration, and (iv) blood circulation. Since nanoparticles are smaller, it is easier for them to quickly penetrate deeper into the respiratory system after being inhaled. The process of absorption also allows nanoparticles to enter the dermal system through wound or abrasion skin where particles smaller than 5 nm diffuse into the skin easily. Thus, entered nanoparticles travel throughout the bloodstream, interact with the systems/organs and accumulate, which could cause severe impact on organs including the lung, liver, reproductive system, and kidneys [21]. Numerous nanoparticles have been shown to have the potential to overcome biological boundaries and injure important organs like the kidney, brain, and liver [33,34]. These nanoparticles could even reach the reproductive system where there are more investigations done on *in vitro* and *in vivo* models for studying the medical and molecular effects of nanotoxicity on genital organs [33]. As discussed in the previous section, these nanoparticles tend to cause cytotoxicity in the genetic and molecular levels by promoting inflammation, apoptosis, and oxidative stress through ROS [34,35]. Nanoparticles reach and accumulate in reproductive organs by breaching the guard reproductive tissues including epithelial, placental, and blood-testis barriers and by destroying Leydig cells, germ cells, and Sertoli cells. This accumulation does harm the male including epididymis and testes and impairs the quality, quantity, motility, and shape of sperm. Female reproductive organs like the ovary and uterus showed a reduction in the number of mature oocytes and interfered with the growth of primary and secondary follicles on the entry of nanoparticles [17,34].

In the male reproductive system, nanomaterials trigger cell death after penetrating through the blood testis barrier (BTB). It results in the lowering of mobility of the sperm due to mitochondrial dysfunction. Its transmission depends upon the polarity, shape, and size of the nanoparticle [33,34,35,36]. According to recent studies, nanoparticles can be endocytosed by granulosa and thecal cells, which stops oocyte development *in vivo* and causes aberrant hormone release [37]. The most common nanoparticles involved in studies assessing their toxicity to female reproductive systems are carbon nanoparticles, metal, metal oxides, and quantum dots. Studies conducted in both *in vivo* and *in vitro* conditions revealed that specific sizes of nanoparticles could enter and accumulate in different female germ cells, triggering a variety of cellular reactions including oxidative stress, signal transduction inhibition, apoptosis, DNA damage, and inflammation [38].

## 4. Impact on the Male Reproductive System

Both oxidative stress and inflammation are believed to be sensitive to the male reproductive system; where nanoparticles’ exposure readily increases oxidative stress and causes cell death and poor spermatogenesis, oxidative stress is reported to be the primary cause of 30–80% of infertility problems in male [39,40]. Studies suggest that nanoparticles react to distinct germ cells and damage cells differently. The changes of the cytoskeleton on the entry of nanoparticles can affect the production of sperm flagella and their ability to migrate, as well as the formation of Sertoli cell tight junctions and the tight junctions between them; all these changes can affect spermatogenesis [41]. Liu et al. [42] have proposed that ZnO nanoparticles caused down-regulation of tight junction proteins in Sertoli cells resulting in BTB dysfunction and no changes in cytoskeleton dynamics were identified [42]. The various nanoparticles involved in the toxicity of the male reproductive system are tabulated in Table 1.

The buildup of nanoparticles destroys germ cells, Leydig cells, and Sertoli cells, impairing the motility, shape, quality, and quantity of the sperm and limiting the number of mature oocytes or preventing the growth of primary and secondary follicles. Further, nanoparticles can alter the number of hormones released, altering sexual behavior [34]. In a study, Mathias et al. [43] found there were no differences in any of the sexual activities in silver nanoparticles on the following sexual behaviors like number of mounts, intromissions, ejaculatory intervals, attempted mounts, and ejaculations, but the exposure to nanoparticles was reducing sperm quality. Nanoparticles such as silica nanoparticles were found to increase the levels of micronucleus frequencies, malondialdehyde levels, and lower activity of catalase and glutathione content in testicular tissues at a higher concentration treated group, pointing to mechanisms of DNA damage and oxidative stress. Significant testicular histological changes were also seen in this group along with inflammation, testicular apoptosis, and oxidative stress by enhancing the gene expression corresponding to the pro-inflammatory activity, apoptotic activity, and oxidative stress caspase 3 and iNOS [44].

Metal nanoparticles like zinc oxide nanoparticles (ZnO) are observed to be breaking the cell membrane and outer membrane of mitochondria in Sertoli cells and down-regulating the production of gap junction proteins, thus damaging the BTB, further compromising its integrity. Additionally, ROS and cytokine release play substantial roles in BTB disruption, markedly raising the oxidative stress status by causing elevated ROS and malondialdehyde levels and lowered glutathione levels and raising the TNF-α levels in Sertoli cells [42]. Similarly, Bara and Kaul [45] have observed that ZnO nanoparticles considerably lowered the expression of the antioxidant enzyme gene (SOD) and significantly enhanced the expression of genes associated with steroidogenesis by the up-regulation of steroidogenic acute regulatory protein and cytochrome P450 side-chain cleavage enzyme in the mouse model. In contrast, exposure to ZnO nanoparticles markedly boosted testosterone synthesis at a concentration level of 2 mg/mL [45].

Likewise, the subcutaneous injection of titanium oxide nanoparticles in pregnant mouse found to have reduced sperm production, disordered seminiferous tubules and olfactory bulb apoptosis was observed [46]. Some of the studies have been done on the estimation of the levels of endocrine and reproductive hormones such as follicle-stimulating hormone, testosterone, luteinizing hormone, estradiol, and gonadotropin-releasing hormone that can be related to the rise in ROS and the concurrent decline in antioxidant enzymes. The sex hormone profile might also be disturbed by titanium oxide nanoparticles’ administration, as seen by lower blood testosterone levels and higher serum levels of estradiol, luteinizing hormone, and follicle-stimulating hormone [47,48]. According to prior research using mouse, rat, and porcine Leydig cells, the inhibitory effect of TNF-α on testosterone production may be caused by a decrease in the 17 α-hydroxylase/C17-20 lyase gene and protein expression and cholesterol side-chain cleavage enzyme, two essential enzymes in testosterone biosynthesis [49]. Contradictory reports are also there, which are as follows: Lauvås et al. [50] and Ogunsuyi et al. [51] found titanium oxide nanoparticles do not cause changes in testosterone levels but according to Miura et al. [52], titanium oxide nanoparticles did not impact sex hormones related to spermatogenesis but they do report that titanium nanoparticles tend to reduce testosterone levels and sperm mobility.

Cerium oxide nanoparticles were found to influence prepubertal spermatogenesis and harm Sertoli cells. Also, lowered expression of the steroidogenic enzyme’s genes *Cyp17-1* and *HSD3b1* is associated with decreased expression of *Insl3*, a gene that specifically marks Leydig cells [53]. There are studies where the Superparamagnetic Iron Oxide Nanoparticles (SPIONs), gold and silver nanoparticles reduce the earthworm in number, but the direct interaction with the reproductive system is not clear [54,55,56,57,58].

**Table 1:** Various nanoparticles are involved in the toxicity of the male reproductive system.

S. No	Nanoparticles	Animal Model	Toxicity Effect	References
1.	Silica nanoparticles	Male Albino Rats	Higher levels of micronucleus frequencies and malondialdehyde levels, and lesser catalase and glutathione activity in testicular tissues.	[44]
2.	ZnO nanoparticles	TM-4 Sertoli cell line and GC2-spd spermatocyte cell line of mouse	Breakdown of the cell membrane and outer membrane of mitochondria in Sertoli cells; down-regulating the production of gap junction proteins; disruption of BTB disruption.	[42]
		Mouse testis Leydig cells	Decreased antioxidant enzyme gene expression (SOD) and increased steroidogenesis-related gene expression.	[45]
3.	Titanium oxide nanoparticles	Pregnant mouse model	Apoptosis of the olfactory bulb occurs with decreased sperm production and motility; disordered and disrupted seminiferous tubules.	[46]
		Mouse, rat, and porcine Leydig cells	17–α hydroxylase/C17-20 lyase and cholesterol side-chain cleavage enzyme gene and protein expression are affected by TNF- α to decrease testosterone synthesis.	[49]
4.	Cerium oxide nanoparticles	Pregnant mouse model	Involvement in the prepubertal spermatogenesis and germ cell; reduction of germ cells, deformation of Sertoli cells; impairment steroidogenesis.	[53]

### Spermatogenesis and Toxicity

**Figure 3:**
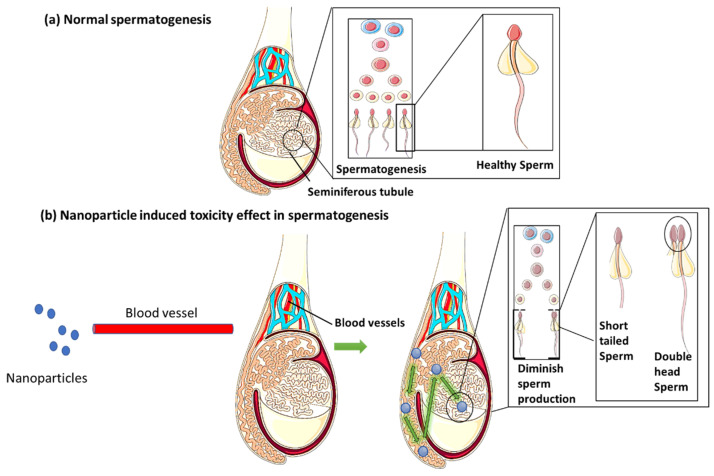
Comparison between normal spermatogenesis and nanotoxicity effect induced spermatogenesis in the male reproductive system (Figure was drawn using the images and illustrations available on Smart Servier Medical Art).

According to some of the research, nanoparticles are found to have the ability to quickly pass the BTB, after building up in the testis and having a negative impact on spermatogenesis. ROS generation in the seminiferous tubule, the place where spermatogenesis takes place, can cause DNA damage to spermatogenic cells [59]. While the testicular tissues are exposed to nanoparticles, seminiferous tubules undergo histological changes, which damage the testicles and diminish sperm production [40] (Figure 3).

Nanoparticles are proven to enter the male rat reproductive organs or tissues in various ways; the testes and the epididymis are considered to be the most vulnerable to damage. In a study, it has been proposed that nanoparticles may affect the testes in a variety of ways, resulting in differences in the production of sperm quantity and quality production. Male mouse testes exposed to water-soluble carbon nanotubes showed oxidative stress, which reduced spermatogenic epithelium thickness [8]. Subcutaneous exposure of silver nanoparticles in male rats displayed abnormalities in the testes as well as changes in sperm quantity and motility, as well as levels of testosterone, luteinizing hormone, and follicle-stimulating hormone. While some studies claim that nanoparticles cannot permeate the skin, others have found evidence of metallic nanoparticles, especially iron nanoparticles, penetrating the skin through hair follicles [60]. Mice exposed to iron oxide nanoparticles caused histopathological changes in seminiferous tubules of the testes including sloughing, detachment, and vacuolization [61]. Exposure to silver nanoparticles resulted in various sperm cell irregularities, such as multiple heads, lengthy tails, and hook attachment errors [62]. Additionally, ultrastructural changes in spermatogonia, spermatogenic cells, and Sertoli cells, as well as atrophy in seminiferous tubules, necrosis, and cell disintegration with abnormal development of spermatids were observed [63].

Also, non-metal nanoparticles such as carbon nanotubes were reported to disrupt the Leydig and Sertoli cell function in the testes, which may lead to a variety of problems in steroidogenesis and germ cell differentiation. Carbon nanotubes might also prevent BTB and hemato-testicular barrier (HTB) from functioning properly [64]. These barriers are permeable to carbon nanotubes, which therefore have a direct impact on the neuroendocrine pathways, spermatogenesis, and reproductive organs [65]. They may have an impact on the level of sex hormones in the blood and the serum or tissue level of endocrine hormones in carbon nanotubes [64]. In a study, ROS levels and biomarkers indicative of oxidative stress in the testes are measured to better understand the mechanism underlying the oligospermia and teratozoospermia brought on by silica nanoparticles. The results have revealed that silica nanoparticles significantly increased the ROS level and malondialdehyde as well as the activity of superoxide dismutases. As a result, it has been hypothesized that alterations in the redox system and sex hormones might be the reason for the lower sperm quality and quantity [66].

Administration of quantum dots to mice was found to decrease the follicle-stimulating hormone and testosterone and a dose-dependent increase in the toxicity of quantum dots on spermatogenesis occurred, and it lasted for about 60 days. Additionally, approximately 60% of pachytene spermatocytes showed unrepaired double-strand break at days 14 and 30 after injection and were found to be vanished at day 60 indicating that double-strand break repair was impaired after exposure to quantum dots. The quantum dots-treated groups were still observed to have a lot of *H2AX* foci in the pachytene stage, and during meiosis I, the fraction of spermatocytes in the pachytene stage was much higher than expected on exposure [67]. Hong et al. [68] have demonstrated that mice exposed to titanium nanoparticles had lesions of the testes and epididymis, experienced reductions in the concentration and motility of sperm, and produced a greater number of defective sperm. Additionally, in mice’s testes, exposure to titanium nanoparticles increased the activities of testicular-marked enzymes such as alkaline phosphatase, acid phosphatase, and total nitric oxide synthase while decreasing the activities of succinate dehydrogenase, sorbitol dehydrogenase, lactate dehydrogenase, and glucose-6-phosphate dehydrogenase. Additionally, exposure to titanium nanoparticles increased the generation of ROS, malondialdehyde, a product of lipid peroxidation, carbonyl, a product of protein oxidation, and 8-hydroxydeoxyguanosine, a product of DNA oxidation in the testes. Also, a reduction in sperm count and lesions induced by titanium nanoparticles was observed [68].

## 5. Impact on the Female Reproductive System

**Figure 4:**
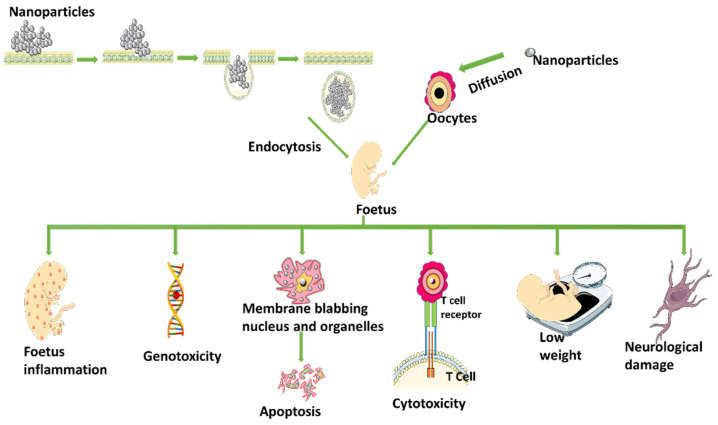
Entry of nanoparticles into foetus and its adverse effect (Figure was drawn using the images and illustrations available on Smart Servier Medical Art).

Nanoparticles alter sex hormone levels by triggering secretory cells such as thecal cells, follicle cells, granule cells, and corpus luteum via the hypothalamic pituitary gonadal axis, or by directly stimulating secretory cells such as granule cells, thecal cells, follicle cells, and the corpus luteum [38]. Some nanoparticles can reach the foetus by passive diffusion or endocytosis, causing fetal inflammation, apoptosis, genotoxicity, reproductive deficit, lower weight, cytotoxicity, immunodeficiency, and neurological damage, among other effects [38] (Figure 4). The primary female sex hormones in females are estrogen and progesterone, which are mostly produced in the ovaries or placenta during pregnancy in humans. Some data suggest that certain nanoparticles can change the gene expression that encodes proteins involved in steroidogenesis, such as ovarian genes essential for the synthesis of estrogen and/or progesterone [32]. The various nanoparticles involved in the toxicity of the female reproductive system are given in Table 2.

**Figure 5:**
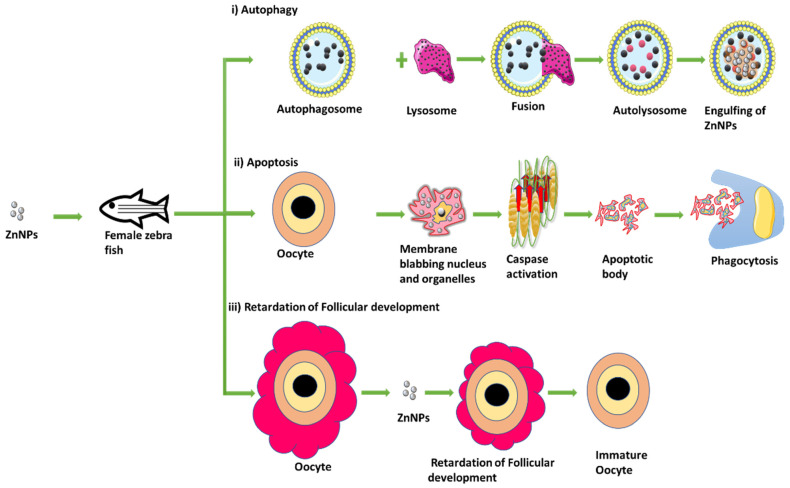
ZnO nanoparticles inducing oxidative stress by autophagy, apoptosis, and retardation of follicular development (Figure was drawn using the images and illustrations available on Smart Servier Medical Art).

In a study, it has been proposed that ZnO nanoparticles have triggered autophagy and apoptosis in a caspase-dependent manner and induced oxidative stress by raising the level of the mutant ovarian p53 protein in maturing oocytes. Necroptosis, having the characteristics of both necrosis and apoptosis, have also been discovered where ZnO nanoparticles produced a necrotic environment that was conducive to retardation of follicular development, changed ovulation of oocyte, and decreased female zebrafish fertility [69] (Figure 5). In a pregnant mouse model, metal ions such as cadmium oxide nanoparticles are found to delay weight gain, decrease the weight of the placenta, and increase the weight of the uterus. Additionally, noticeable changes in the number of estrogen receptors α and β expression in uterine tissues finally led to a decrease in implantations. The release of cadmium ions from cadmium oxide nanoparticles has the potential to disrupt and unbalance the blastocyst before implantation as well as cause endocrine disturbance to prevent it [70,71].

The toxicity of the titanium oxide nanoparticles was found to be associated with *Cyp17a1*, a gene that regulates the secretion of the hormone, which was up-regulated, indicating enhanced estradiol production. Genes, including *bmf*, were up-regulated while some of the genes that control apoptosis were down-regulated. Changes in the ovary’s immunological and inflammatory responses, oxidoreductase activity, oxidative stress, transcription, cell proliferation, and ion transport were also noted [72]. The Zona Pellucida (ZP) of oocytes was found to be accumulated by cerium oxide nanoparticles in follicular cells through endocytosis. Follicular cell endocytosis and zona pellucida trapping could not able to shield mature oocytes from oxidative stress and DNA damage when exposed to high concentrations [73]. Mouse oocytes cultivated in media containing cerium oxide nanoparticles at a lower concentration (0.01 mg/L) during *in vitro* fertilization (IVF) had a considerably decreased fertilization rate compared to the control group. Low fertility rates could be caused by gamete genotoxicity and oxidative stress brought on by cerium oxide [38,74]. Silver nanoparticles have decreased the primary oocytes which resulted in the influence and inhibition of ovulation by entering the ovaries after entering the circulation and penetrating cells to cause oxidative stress, which activates oxidative stress factors in ovarian cells and causes apoptosis. Hence, it can be concluded that silver nanoparticles, at various concentrations, could cause oxidative stress by increasing degeneration in primary oocytes associated with lower antioxidant status [75].

**Table 2:** Various nanoparticles are involved in the toxicity of the female reproductive system.

S. No.	Nanoparticles	Animal Model	Toxicity Effect	References
1.	ZnO nanoparticles	Female zebrafish	Autophagy and apoptosis occurring in a caspase-dependent manner; increased oxidative stress by inducing mutant ovarian p53 protein; necroptosis; follicular developmental retardation; deformation of oocyte ovulation, and decreased female zebrafish fertility.	[69]
2.	Cadmium oxide nanoparticles	Pregnant mouse model	Weight gain, increased uterus weight, and decreased weight of placenta; decreased quantity of estrogen receptors.	[70]
3.	Titanium oxide nanoparticles	Female mice	Up-regulation of *Cyp17a1* resulted in enhanced estradiol production; up-regulation of *bmf* genes; apoptotic genes were down-regulated.	[72]
4.	Cerium oxide nanoparticles	Mouse oocytes	Accumulation in the zona pellucida (ZP) of oocytes; DNA damage due to follicular cell endocytosis and zona pellucida trapping.	[73]
5.	Silver nanoparticles	Ovaries of female albino rats	Inhibition of the ovulation; activation of oxidative stress factors in ovarian cells resulting in apoptosis.	[75]

### Nanotoxicity on the Steroidogenic Pathway

The potential harmful effects of nanoparticles such as induction of cellular states of oxidative stress, modulation of enzyme activity, inflammation, and cell death include disruption of gonadal steroidogenesis-related biochemical and physiologic processes [16]. Larson et al. [76] reported that gold nanoparticles could be a new class of ovarian endocrine-inhibiting compounds if exposed for extended periods or at high concentrations as they disrupt the production of sex-steroid hormones, resulting in reproductive problems in humans and animals [76]. By contrast, *Gallus domesticus* treated with silver nanoparticles showed no significant differences in progesterone levels between the granulosa and theca layers, but a progressive reduction in estradiol and testosterone levels was observed in theca cells. [77]. In preovulatory rat granulosa cells, multi-walled carbon nanotubes (MWCNTs) prevented the synthesis of progesterone. Particularly, at concentrations of 10 and 50 g/mL/48 h, the production was drastically reduced. Expression of Steroidogenic Acute Regulatory protein (StAR), a steroidogenic protein that helps in the movement of cholesterol from the outer to the inner mitochondrial membrane, where it is processed by P450scc to pregnenolone, was also altered by MWCNTs [78,79]. Other non-metal nanoparticles such as calcium phosphate nanoparticles have induced apoptosis and have no effect on S phase cell cycle arrest, progesterone level, estradiol levels, and the mRNA levels of P450scc, and StAR was observed in human ovarian granulosa cells cultured in-vitro [80]. Similarly, nano-silica particles caused fetotoxicity and placental dysfunction in foetus placenta of pregnant mice [81]. The decreased activity of the steroidogenic enzymes 3β and 17β-hydroxysteroid dehydrogenase affected gonadal steroidogenesis in fullerene-treated female *Anabas testudineus*. Serum cortisol levels significantly increased whereas estradiol levels in female fish significantly decreased. There was a significant difference in alkali-labile phosphate levels, plasma calcium, and total protein among female and male fish, possibly due to C_60_ fullerene antiestrogenic properties. In the ovaries and brain of female fish, aromatase enzyme activity considerably decreased [80].

## 6. Nanotoxicity Quantification Tests

The presence of nanotoxicity in the reproductive system can be identified by the collection of test samples and subjecting them to the following tests. These include tests for genotoxicity, proliferation, and mutated gene expression in cell culture as well as tests for cytotoxicity-altered metabolism, reduced growth, or lytic or apoptotic cell death [82].

### 6.1. Assay for the Determination of ROS Production Due to Oxidative Stress

Nanoparticles entering the cell may cause oxidative stress to the cell leading to the production of reactive oxidative species such as superoxide, α-oxygen, hydroxyl radical, peroxides, singlet oxygen, etc. The defense mechanisms of ROS production are of two types: enzymatic and non-enzymatic scavengers. Enzymatic scavengers include catalase, glutathione peroxidase, and superoxide dismutase, whereas non-enzymatic scavengers are glutathione, melatonin and vitamin A, C, and E [83]. These biomarkers act as indicators for biological, pathogenic processes, therapeutic, or pharmacological responses [84].

#### 6.1.1. Superoxide Dismutase (SOD) Assay

Superoxide dismutases (SODs) are a crucial antioxidant defense system in the body that protects against oxidative damage. Reactive oxygen species-related disorders can be treated effectively with the enzyme. [85]. It is involved in the conversion of hydrogen peroxide and molecular oxygen from superoxide radicals [86]. SOD is the first line of defense mechanism against ROS in live cells by accelerating this conversion by redox disproportionation [87]. The assay measures the activity of superoxide dismutase in the mitochondria of the cell helping in the quantification of stress formed in the cell. In order to identify the presence of SOD by reaction with INT (2-(4-iodophenyl)-3-(4-nitrophenol)-5-phenyltetrazolium chloride) and formation of red formazan. SOD present in the sample prevents the conversion of superoxide radicals into oxygen. Further, the absorbance was observed at 505 nm. The SOD activity of the sample was estimated using the standard curve drawn using SOD enzyme as the standard solution [88].

#### 6.1.2. Catalase Assay

Hydrogen peroxide (H_2_O_2_), a non-radical ROS, is the primary substrate for catalase, an essential enzyme. It is responsible for breaking down hydrogen peroxide and neutralizing it, as well as maintaining the required level of the molecules in the cell for cellular signalling [89]. The catalase activity can be determined by the conversion of cobalt (II) to cobalt (III) using H_2_O_2_ in the presence of bicarbonate solution. The formation of the complex carbonato-cobaltate (III) complex ([Co(CO_3_)_3_]Co) is used for the determination of catalase enzyme activity in the sample, where sample is added with 10 mM hydrogen peroxide and incubated at the temperature of 37 °C for 2 min. Then the mixture solution containing cobalt (II), sodium bicarbonate, Graham salt solution is added. This was then vortexed for 5 sec and then kept at room temperature for 10 min [90] the absorbance of the standard solution (mostly 10mM hydrogen peroxide and not added with sample) and sample are taken at 440 nm and compared [90].

#### 6.1.3. Glutathione Peroxidase Assay

Glutathione peroxidase (GPx) is a cytosolic enzyme that is responsible for reducing hydrogen peroxide to water and oxygen as well as reducing peroxide radicals to alcohols and oxygen [91]. It is a qualitative reaction of tert-butyl and cumene hydroperoxides and glutathione transferase which helps in the evaluation of peroxidase activity. It is estimated by measuring the reduction of H_2_O_2_ by glutathione peroxidase to alcohol through NADPH loss. The gel containing glutathione is run with the sample, further incubated in 0.008% of cumene hydroperoxide for 10 min and then stained using 1% ferric chloride (FeCl_3_) and 1% potassium ferricyanide (C_6_N_6_FeK_3_). The formation of achromatic bands indicates the presence of glutathione peroxidase [86].

### 6.2. Other Methods

Various biological specimens (serum, urine, plasma, follicular/peritoneal/seminal fluid) can be used to assess oxidative stress, enabling accurate evaluation of redox status and the planning of therapeutic antioxidant supplements, if necessary [92]. Oxidative stress can be measured by the identification of the modification of protein, lipid, and DNA. The lipid modification can be identified by the byproduct of malondialdehyde, 4-hydroxy 2-nonenal, 4-oxonon-2-enal, and acrolein; for protein, dityrosine is used as biomarker, and for DNA, 8-hydroxy-2′-deoxyguanosine (8-oxodG) is used as biomarker, which is measured using ELISA [93]. Numerous techniques are used to quantify ROS in semen, including (i) chemiluminescence, (ii) cytochrome c reduction test, (iii) nitro blue tetrazolium test, and (iv) electron spin resonance. Various other methods include (i) fluorescent anisotropy which assesses membrane fluidity sperm motility defects, (ii) oxidation-reduction potential (ORP) measurement, which detects oxidative stress in seminal fluid, and (iii) Oxygen Radical Absorbance Capacity (ORAC) Assay, (iv) ALDETECT Assay [92]. Oxidative stress in the female reproductive system can be evaluated using common biomarkers like SOD, GPx, oxidative DNA adducts, conjugated dienes, thiobarbituric acid reactive substances, lipid peroxides, reverse transcription-polymerase chain reaction, enhanced chemiluminescence assay, immunocytochemical staining, etc [94].

## 7. Role of Antioxidants in Nanoparticle-Induced Stress

Any agent or compound that could prevent the oxidation of a suitable substrate even at low concentrations is referred to as an antioxidant. These are stable compounds that can give an undesired free radical species an electron, neutralize it, and reduce its destructive potential. Generally, the scavenging abilities of these antioxidants either prevent or delay cellular damage. These antioxidants’ low molecular weights make it simple for them to engage with ROS (i.e., free radicals) and stop their chain reaction before they damage important molecules involved in the normal functioning of the cells [95]. Some of the naturally occurring antioxidants mostly obtained from plants [96,97,98,99] have the ability to strengthen antioxidant defense mechanisms and prevent and reduce organism damage brought on by oxidative stress produced by nanoparticles. In particular, they play a key role in the prevention, treatment, and control of nanoparticle-induced toxicity and oxidative stress [100]. Vitamin C, sometimes referred to as ascorbic acid, is an antioxidant that can scavenge free radicals. The formation of ROS by the silver nanoparticles is completely reduced by the addition of ascorbic acid. It concurrently reduces DNA damage, apoptosis, and mitochondrial damage brought on by silver nanoparticles, lessening their harmful effects [15]. One naturally occurring flavonoid found in numerous plants and foods, quercetin, is an antioxidant with the capacity to scavenge free radicals. By promoting bad phosphorylation and translocation of *Nrf2* through PI_3_-K/Akt-dependent pathways, quercetin has been demonstrated to lessen oxidative damage and inflammation brought on by Fe_2_O_3_ nanoparticles [101].

Lycopene has been shown to control the activity of redox-sensitive molecular targets, including the mitogen-activated protein kinases and *Nrf2* activation [102]. In a study, rats receiving lycopene supplements had higher levels of expression of *Nrf2*, *HO-1*, glutathione, and antioxidant enzymes such as catalase, superoxide dismutase, and glutathione peroxidase, thus increasing antioxidant activity against oxidative stress [103]. Also, diterpenes from the leaves of *Stevia rebaudiana* reduce the proinflammatory cytokines production (TNF-α, IL-1β, and IL-6) by altering the I- κB/NF- κB pathway [104]. The ROS- mediated lethal toxicity of the ZnO nanoparticles and titanium oxide nanoparticles was found to be scavenged by curcumin and vitamin C in *Caenorhabditis elegans* (*C. elegans*) [105]. Some of the other findings have reported that the antioxidants such as vitamin E and anti-amyloid compounds glycyrrhizic acid significantly reduce the effects of nano-aluminum oxide-induced oxidative stress, graphene oxide nanoparticles-induced toxicity, and nano-silica-induced inhibition of serotonin neurotransmission in *C. elegans* [106,107,108].

Recently, antioxidants in nanoparticle form have been suggested as an original way to enhance their properties [23]. Several nanoparticles made of biologically derived compounds with antioxidant properties have been discovered where loading or tagging of bioactive compounds is possible [23,96,97]. An innovative and potent replacement could be phyto-antioxidant functionalized nanoparticles. In addition to serving their original purpose, they can offer oxidative damage protection [109]. Copper nanoparticles synthesized using *Hibiscus rosa-sinensis* showed great scavenging of H_2_O_2_ and Ferric-Reducing Antioxidant Power (FRAP), demonstrating a strong antioxidant activity [110]. Similarly, copper nanoparticles made from *Dioscorea bulbifera* tubers (DBTE) revealed scavenging activity against nitric oxide, superoxide radicals, and 1,1-diphenyl-2-picrylhydrazyl (DPPH). This demonstrated the importance of copper nanoparticles in the reduction of oxidative stress [111]. Silver nanoparticles made from *Costus* leaves had antioxidant activity comparable to that of ascorbic acid with an IC_50_ value of less than 50 mg/L, making them more potent DPPH scavengers than leaf extract of *Costus* [112]. Similarly, silver nanoparticles made from *Cestrum nocturnum* leaves tested for antioxidant activity were discovered to have effective DPPH scavengers with a percentage of 29.55% at the concentration of 100 μg/mL rather than ascorbic acid with a percentage of 24.28% at the same dosage [113]. Also, the recent development of ROS-responsive drug delivery systems such as sulfur-containing polymers, thioether-containing polymers, poly(thioketal), selenium-containing polymers, etc., act by breaking the chemical bonds and/or transitions from hydrophobic to hydrophilic phases, favoring the release of carrier medicines helping in the treatment of ROS [114].

## 8. Conclusions

The reproductive system is now being exposed to more nanoparticles due to the increased advancement in nanotechnology. Male and female reproductive systems have been reported to be negatively affected by a number of nanoparticles. The transmission of genetic and epigenetic information to subsequent generations is carried out by germ cells, which serve as a link between generations. It has been addressed how different nanoparticles can be hazardous to the reproductive system. Even though it is unavoidable and has been determined to have a slower effect on people, animals, and the environment, careful consideration of the effects and toxicity of nanoparticles is essential.

## Data Availability

The data used to support the findings of this study are included in the article. Should further data or information be required, these are available from the corresponding author upon request.
